# Renal and Vascular Effects of Combined SGLT2 and Angiotensin-Converting Enzyme Inhibition

**DOI:** 10.1161/CIRCULATIONAHA.122.059150

**Published:** 2022-07-11

**Authors:** Yuliya Lytvyn, Karen Kimura, Nuala Peter, Vesta Lai, Josephine Tse, Leslie Cham, Bruce A. Perkins, Nima Soleymanlou, David Z.I. Cherney

**Affiliations:** Department of Medicine, Division of Nephrology, Toronto General Hospital (Y.L., V.L., J.T., L.C., D.Z.I.C.); Temerty Faculty of Medicine (Y.L.); Department of Medicine, Division of Endocrinology and Metabolism, Mount Sinai Hospital, University of Toronto, Canada (B.A.P.).; Boehringer Ingelheim Canada Ltd/Ltée, Burlington (K.K.).; Biberach an der Riss, Germany (N.P.).; Boehringer Ingelheim Pharmaceuticals Inc, Ridgefield, CT (N.S.).

**Keywords:** angiotensin-converting enzyme inhibitors, glomerular filtration rate, hemodynamics, sodium-glucose transporter 2 inhibitors

## Abstract

The cardiorenal effects of sodium-glucose cotransporter 2 inhibition (empagliflozin 25 mg QD) combined with angiotensin-converting enzyme inhibition (ramipril 10 mg QD) were assessed in this mechanistic study in patients with type 1 diabetes with potential renal hyperfiltration.

**Methods:**

Thirty patients (out of 31 randomized) completed this double-blind, placebo-controlled, crossover trial. Recruitment was stopped early because of an unexpectedly low proportion of patients with hyperfiltration. Measurements were obtained after each of the 6 treatment phases over 19 weeks: (1) baseline without treatment, (2) 4-week run-in with ramipril treatment alone, (3) 4-week combined empagliflozin-ramipril treatment, (4) a 4-week washout, (5) 4-week combined placebo-ramipril treatment, and (6) 1-week follow-up. The primary end point was glomerular filtration rate (GFR) after combination treatment with empagliflozin-ramipril compared with placebo-ramipril. GFR was corrected for ramipril treatment alone before randomization. At the end of study phase, the following outcomes were measured under clamped euglycemia (4 to 6 mmol/L): inulin (GFR) and para-aminohippurate (effective renal plasma flow) clearances, tubular sodium handling, ambulatory blood pressure, arterial stiffness, heart rate variability, noninvasive cardiac output monitoring, plasma and urine biochemistry, markers of the renin-angiotensin-aldosterone system, and oxidative stress.

**Results:**

Combination treatment with empagliflozin-ramipril resulted in an 8 mL/min/1.73 m^2^ lower GFR compared with placebo-ramipril treatment (*P*=0.0061) without significant changes to effective renal plasma flow. GFR decrease was accompanied by a 21.3 mL/min lower absolute proximal fluid reabsorption rate (*P*=0.0092), a 3.1 mmol/min lower absolute proximal sodium reabsorption rate (*P*=0.0056), and a 194 ng/mmol creatinine lower urinary 8-isoprostane level (*P*=0.0084) relative to placebo-ramipril combination treatment. Sodium-glucose cotransporter 2 inhibitor/angiotensin-converting enzyme inhibitor combination treatment resulted in additive blood pressure–lowering effects (clinic systolic blood pressure lower by 4 mm Hg [*P*=0.0112]; diastolic blood pressure lower by 3 mm Hg [*P*=0.0032]) in conjunction with a 94.5 dynes × sex/cm^5^ lower total peripheral resistance (*P*=0.0368). There were no significant changes observed to ambulatory blood pressure, arterial stiffness, heart rate variability, or cardiac output with the addition of empagliflozin.

**Conclusions:**

Adding sodium-glucose cotransporter 2 inhibitor treatment to angiotensin-converting enzyme inhibitor resulted in an expected GFR dip, suppression of oxidative stress markers, additive declines in blood pressure and total peripheral resistance. These changes are consistent with a protective physiologic profile characterized by the lowering of intraglomerular pressure and related cardiorenal risk when adding a sodium-glucose cotransporter 2 inhibitor to conservative therapy.

**Registration:**

URL: https://www.clinicaltrials.gov; Unique identifier: NCT02632747.

**Clinical Perspective**

**What Is New?**

- We demonstrated that the addition of empagliflozin to ramipril for 4 weeks improves surrogate measures of renoprotection in people with uncomplicated type 1 diabetes and preserved kidney function.
- Physiologic mechanisms of sodium-glucose cotransporter 2 inhibitors hypothesized to modulate tubuloglomerular feedback and reduce glomerular hypertension were ameliorated with the addition of empagliflozin, represented by a decreased proximal renal tubular sodium reabsorption and an anticipated dip in glomerular filtration rate.
- Empagliflozin, when added to ramipril, lowered blood pressure and total peripheral resistance without affecting arterial stiffness, heart rate variability, or cardiac output, suggesting a novel sodium-glucose cotransporter 2 inhibitor–mediated antihypertensive mechanism.

**What Are the Clinical Implications?**

- This is the first mechanistic trial that evaluated the effect of adding sodium-glucose cotransporter 2 to angiotensin-converting enzyme inhibition on physiologic effects, including acute effects on glomerular filtration rate, which were consistent with cardiorenal protection in clinical trials that have to date only included individuals with type 2 diabetes or nondiabetic kidney disease.
- Given the consistency of physiologic effects across different cohorts, including in patients with type 1 diabetes, future clinical trials should continue to evaluate whether chronic sodium-glucose cotransporter 2 inhibition reduces the risk of cardiorenal complications in novel populations at high risk of kidney failure or cardiovascular disease.

**Editorial, see p 463**

The discovery that sodium-glucose cotransporter 2 (SGLT2) inhibitors (SGLT2i) reduce the risk of cardiovascular and kidney disease in patients with type 2 diabetes (T2D) has had a substantial effect on clinical practice. In cardiovascular outcome trials, these therapies reduced major adverse cardiac events, hospitalization for heart failure and kidney composite end points in patients with T2D,^1^ and cardiorenal end points in patients with albuminuric chronic kidney disease.^2,3^ SGLT2i reduced glycated hemoglobin (HbA1c) by 0.7% to 1.0%, systolic blood pressure (SBP) by 3 to 5 mm Hg, and body weight by 2 to 4 kg.^4,5^

We previously demonstrated that SGLT2i alone exhibit similar effects on glycemic control, body weight, and blood pressure in patients with uncomplicated type 1 diabetes (T1D), a cohort that models early cardiorenal physiologic changes in patients with diabetes at a stage before the development of clinical complications.^6^ Moreover, kidney hyperfiltration is significantly attenuated with SGLT2i monotherapy.^7^ In light of the poor prognosis associated with hyperfiltration^8,9^ and kidney protective effects of SGLT2i in experimental models of T1D,^10,11^ these findings suggest that SGLT2i may promote a protective decrease in intraglomerular hypertension, even if used before the onset of clinical chronic kidney disease.^12–14^

Whereas the mechanistic basis for glomerular hypertension in diabetes is complex, hyperglycemia plays an important role through neurohormonal activation and via effects on tubular function. First, the neurohormonal hypothesis of hyperfiltration suggests that activation of the renin-angiotensin-aldosterone system (RAAS) during hyperglycemia triggers vasoconstriction at the efferent renal arteriole.^1,5–14^ In addition, according to the tubular hypothesis (Figure 1), hyperglycemia-induced overexpression of SGLT2 at the proximal tubule^15^ increases reabsorption of sodium (Na^+^) and leads to decreased distal Na^+^ delivery to the macula densa. This signal gets incorrectly sensed as a reduction in effective circulating volume leading to vasodilation of the afferent renal arterioles and thus hyperfiltration characteristic of diabetes.^15^ In an 8-week add-on-to-insulin study, empagliflozin induced a reduction in renal hyperfiltration^16^ in young patients with uncomplicated T1D and hyperfiltration that was comparable in magnitude to what has been reported with angiotensin-converting enzyme inhibition (ACEi) in a separate hyperfiltering T1D cohort.^7^ As with RAAS inhibitor (RAASi) monotherapy, SGLT2i did not abolish hyperfiltration, suggesting a potential kidney benefit when these therapies are combined to take advantage of both neurohormonal and tubular pathways. Whereas T1D is a good model of uncomplicated renal hemodynamic changes attributed to diabetes, such mechanisms can also apply to similar renal hemodynamic changes observed in other conditions, such as T2D or obesity.

**Figure 1.**
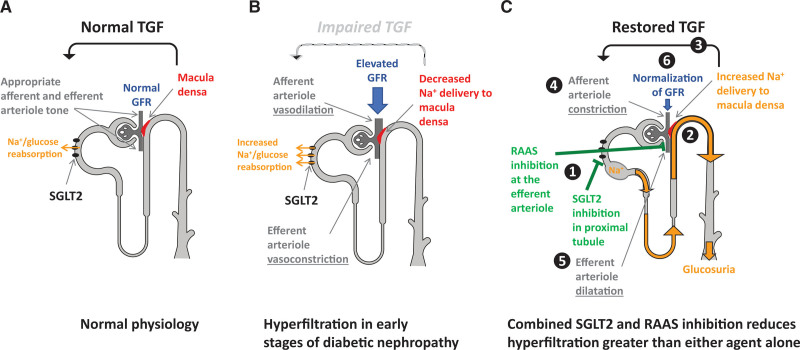
**Postulated mechanisms in normal physiology and hyperfiltration in early stages of nephropathy and after combined inhibition of SGLT2 and RAAS. A**, Under physiologic conditions, tubuloglomerular feedback (TGF) signaling maintains stable glomerular filtration rate (GFR) by modulation of preglomerular arteriole tone. In cases of conditional increases in GFR, the macula densa within the juxtaglomerular apparatus senses an increase in distal tubular sodium delivery and adjusts GFR through TGF accordingly. Neurohormonal signaling using the renin-angiotensin-aldosterone system (RAAS) contributes to maintenance of stable GFR by modulating postglomerular arteriole tone. **B**, Under chronic hyperglycemic conditions during diabetes, increased proximal sodium-glucose cotransporter 2 (SGLT2)–mediated reabsorption of sodium (Na^+^) and glucose impairs this feedback mechanism. Thus, despite increased GFR, the macula densa is exposed to lowered sodium concentrations and results in dilation of the afferent intrarenal arteriole. Increased activation of the renin-angiotensin-aldosterone neurohormonal system in diabetes causes constriction at the efferent intrarenal arteriole. Together, impairment of TGF and neurohormonal signaling likely leads to inadequate arteriole tone at the afferent and efferent arterioles and renal perfusion is increased. **C**, SGLT2 inhibition with empagliflozin treatment blocks proximal tubule glucose and sodium reabsorption, which leads to increased sodium delivery to the macula densa. This condition restores TGF by means of appropriate modulation of arteriolar tone (e.g., afferent vasoconstriction). Blockade of the RAAS with an angiotensin-converting enzyme (ACE) inhibitor, ramipril, leads to efferent vasodilation. The overall effect of afferent vasocontraction with SGLT2 inhibition and efferent vasodilation with ACE inhibition reduces renal plasma flow and hyperfiltration. Modified from Cherney et al.^16^

Our aim was to investigate the additive cardiorenal hemodynamic effects of combined treatment with both agents, specifically neurohormonal suppression (with an ACEi) and activation of tubular (SGLT2i) factors, in patients with uncomplicated T1D, T2D, or obesity as mechanistic models of early cardiorenal physiologic changes before the development of clinical kidney or cardiovascular complications. The primary objective of this clinical trial (BETWEEN Study [Empagliflozin and ACEi- Effects on Hyperfiltration]; URL: https://www.clinicaltrials.gov; Unique identifier: NCT02632747) was to determine the effect of a 4-week treatment with an SGLT2i, empagliflozin 25 mg QD, versus placebo, in addition to an ACEi, ramipril 10 mg QD or maximally tolerated dose, on glomerular filtration rate (GFR). Our secondary objectives were to study the effect of the addition of empagliflozin to ramipril treatment on distal sodium delivery and systemic hemodynamics. We hypothesized that adding empagliflozin treatment to a background of ramipril would decrease GFR and reduce cardiac output and arterial stiffness, leading to declines in SBP.

## Methods

The data, analytic methods, and study materials will be made available to other researchers for purposes of reproducing the results or replicating the procedure.

### Data Sharing Statement

To ensure independent interpretation of clinical study results and enable authors to fulfill their role and obligations under the International Committee of Medical Journal Editors criteria, Boehringer Ingelheim grants all external authors access to relevant clinical study data pertinent to the development of the publication. In adherence with the Boehringer Ingelheim Policy on Transparency and Publication of Clinical Study Data, scientific and medical researchers can request access to clinical study data after the article has been published, regulatory activities are complete, and other criteria are met. Researchers should use the https://vivli.org link to request access to study data and visit https://www.mystudywindow.com/msw/datasharing for further information.

### Study Participants

Thirty participants completed the study and had the primary GFR end point evaluated (Figure S1). Detailed study inclusion and exclusion criteria at screening can be found in Table S1. In brief, inclusion criteria were as follows: male or female patients ≥18 years of age diagnosed with T1D ≥6 months before informed consent, patients with T2D, or obese patients without diabetes; HbA1c of 6.5% to 11.0% for patients with T1D or T2D; estimated GFR ≥60 mL/min/1.73 m^²^; and average blood pressure >90/60 mm Hg and ≤140/90 mm Hg. The main exclusion criteria were as follows: for patients with T1D, treatment with an antihyperglycemic agent within 3 months or history of hypersensitivity; treatment with an SGLT2i within 30 days; severe hypoglycemia that required emergency hospital treatment within 3 months before screening; or occurrence of diabetic ketoacidosis within 3 months. The local Research Ethics Board at the University Health Network (Toronto, Canada) approved the protocol and all participants gave informed consent before start of study procedures. The study was conducted according to the International Conference on Harmonization on Good Clinical Practice.

### Experimental Design

The study was registered at www.clinicaltrials.gov (Unique identifier: NCT02632747). This was a single-center (University Health Network, University of Toronto, Ontario, Canada), prospective, double-blind, randomized, placebo-controlled, crossover study, comprising 6 sequential phases over an ≈19-week time period (Figure S2): (1) a 2-week screening period without treatment; (2) a 4-week run-in period where patients were treated with ramipril 5 mg for 1 week and 10 mg for 3 weeks, following which patients were randomly assigned to follow either sequence A or B; (3) a 4-week period 1, where patients in sequence A were treated with empagliflozin 25 mg QD combined with ramipril 10 mg QD and patients in sequence B were treated with placebo combined with ramipril 10 mg QD; (4) a 4-week washout period where patients were only treated with ramipril 10 mg QD; (5) a 4-week period 2, where patients in sequence A were treated with placebo and ramipril 10 mg QD and patients in sequence B were treated with empagliflozin 25 mg QD and ramipril 10 mg QD; and (6) a 1-week posttreatment follow-up period.

At the completion of the baseline, run-in, and periods 1 and 2, patients underwent full-day cardiorenal physiology assessments under controlled euglycemic clamp conditions (4 to 6 mmol/L) for ≈4 hours preceding and during all investigations (Table S2). During the clamp procedures, blood glucose was maintained at a stable level as described previously.^17^ Clamped euglycemia was maintained to reduce the background glycemic variability and the effect hyperglycemia could have on the outcome measures. For example, acute induction of modest hyperglycemia can induce a hyperfiltration response,^17,18^ raise blood pressure, and influence circulating neurohormonal mediators.^18,19^ Maintaining a physiologic state of euglycemia allowed us to isolate the effects of modulating tubuloglomerular feedback while minimizing neurohormonal activation by systemic hyperglycemia. In normoglycemic, nondiabetic participants, the clamp procedures were not planned as part of the physiologic studies.

Patients remained supine throughout the physiologic assessment visits and during measurements but were allowed to ambulate for voiding. In order to avoid hyperfiltration because of high protein intake or effective circulating volume contraction and RAAS activation from sodium depletion, participants were instructed to adhere to a moderate protein (<1.5 g/kg per day) and high-sodium (>140 mmol/day) diet for 7 days leading up to the 4 physiology assessment visits. Participants were also asked to avoid alcohol and tobacco for at least 48 hours and to fast for a minimum of 12 hours before all physiologic assessment study visits. Treatment compliance was assessed on the basis of tablet count of dispensed and returned medication and was required to be 80% to 120% of the treatment dose.

### Assessment of Renal Hemodynamic Function

After stabilization of the ambient euglycemic clamp, a third intravenous line was used to infuse inulin and para-aminohippurate (PAH). First, the infusion was primed with 25% inulin (60 mg/kg) and 20% PAH (8 mg/kg), followed by a continuous infusion to maintain plasma concentrations at 20 mg/dL and 1.5 mg/dL, respectively. After a 90-minute equilibration period, blood samples were collected for inulin, PAH, and hematocrit with additional blood being drawn 30 minutes later. GFR and effective renal plasma flow (ERPF) were estimated under steady-state conditions of infusing inulin and PAH, respectively.^17,20^ Mean baseline GFR and ERPF values were calculated as a mean of 2 independent clearance periods on each of the physiology assessment visits. Filtration fraction, renal blood flow, and renal vascular resistance (RVR) were calculated as described in Table S3.

### Assessment of Renal Sodium Handling

Tubular sodium handling was assessed using established sodium and lithium clearance techniques.^21,22^ Patients were instructed to take a single lithium carbonate tablet (300 mg) at 22:00 hours (for measurement of fractional lithium excretion) the night before physiologic study visits. Blood and urine samples were collected after euglycemia was achieved during the physiologic study visits for sodium, lithium, and creatinine measurements. The following tubular sodium handling measures were calculated as described in Table S3:^23^ fractional sodium excretion, fractional lithium excretion, absolute proximal fluid reabsorption rate, absolute proximal Na reabsorption rate, fractional proximal fluid reabsorption, distal Na delivery, absolute distal Na reabsorption rate, and fractional distal Na reabsorption.

### Assessment of Cardiovascular Hemodynamic Function and Metabolic Measures

Ambulatory blood pressure monitoring (ABPM) was performed for 24 hours before each physiologic assessment study visit. The ABPM device was programmed to measure blood pressure every 20 minutes throughout the day and night. Mean blood pressure was analyzed during daytime, nighttime, awake time, sleep time, and through to peak time.

Mean arterial pressure, blood pressure, and heart rate were measured by an automated sphygmomanometer over the right brachial artery (Dinamap sphygmomanometer; Critikon) throughout the physiologic assessment study days.

During each physiologic assessment study day, following the glucose clamp, right radial artery and carotid waveforms were recorded with a high-fidelity micromanometer and using the validated transfer function, corresponding central aortic pressure waveform data were generated (SPC-301, Millar Instruments SphygmoCor, AtCor Medical Systems Inc.). Augmentation index, an estimate of systemic arterial stiffness, was calculated as the difference between the second systolic peak and inflection point, expressed as a percentage of the central pulse pressure corrected to an average heart rate of 75 beats per minute. The aortic pulse wave velocity was measured using the same device by sequentially recording ECG-gated right carotid and radial artery waveforms. The average of 2 vascular measurements was reported.

After completion of arterial stiffness testing, heart rate variability testing was performed using AtCor software (AtCor Medical Systems Inc). The average of two 10-minute segments was recorded. Vagal tone (root mean square successive difference) and sympathetic activity (standard deviation of normal-to-normal interval) measures were obtained at each of the 2 periods and the results were then averaged.

Noninvasive cardiac output monitoring (NICOM; Cheetah Medical) was used to measure cardiac output, cardiac power output index, stroke volume, total peripheral resistance, total peripheral resistance index, and thoracic fluid content during each of the physiologic assessment study visits. The NICOM monitoring system is on the basis of bioreactance technology.^24^ Four sensor pads were applied above and below the heart on the chest. Each sensor pad contained an outer transmitting sensor and an inner receiving sensor. The NICOM monitor induced a 75-KHz AC current to the thorax by means of the outer sensors and received the voltage through the inner sensors. The sensors can detect a time delay or phase shift between the induced current and the received voltage. Stroke volume was derived on the basis of consecutive measurements of the phase shift. NICOM measurements were performed for 10 minutes and in duplicate and the mean of the measurements was reported. Weight, waist circumference, fasting plasma glucose, and HbA1c were measured during each physiologic assessment study visit.

### Sample Collection and Analytical Methods for Plasma and Urine Biochemistry Responses

During the steady-state euglycemic clamp conditions, inulin and PAH blood samples were collected to assess renal clearance measures according to standard methods.^20,25^ In addition, blood samples for angiotensin II, angiotensinogen, aldosterone, plasma renin concentration, 8-hydroxydeoxyguanosine, and 8-isoprostane were collected to determine the effect of treatment interventions on these measures.^20,25,26^ At corresponding time intervals, urine samples were collected to assess for nitric oxide (NO), 8-hydroxydeoxyguanosine, 8-isoprostane, and cyclic guanosine monophosphate.^25^ All urinary values were corrected for urinary creatinine at the time of collection and were expressed as a ratio relative to the amount of urinary creatinine excreted at the collection time points. In the 24 hours leading up to physiologic assessments, participants also completed a 24-hour timed urine collection, which was analyzed for volume and glucose excretion.

Plasma renin concentrations were measured with a sandwich chemiluminescence immunoassay kit (LIAISON; DiaSorin SpA). For measurements of 8-hydroxydeoxyguanosine, a Phenomenex Strata-X-A SPE plate, an AB Sciex 5500 Triple Quad spectrometer, and AB Sciex software were used. Aldosterone was measured using chromatography and mass spectrometry and enzyme-linked immunosorbent assay was used to measure urine cyclic guanosine monophosphate, 8-isoporastane nitric oxide, angiotensin II, and angiotensinogen. A 24-hour urine glucose level was assessed by standard laboratory methods. HbA1c was measured by high-performance liquid chromatography.

### Statistical Analyses

The primary end point of this study was GFR after a 4-week treatment with empagliflozin-ramipril combination compared with placebo-ramipril. GFR values were corrected for randomization at the end of the run-in period. The sample size calculation was powered to detect a reduction of GFR with SGLT2i-ACEi by an additional clinically relevant 15 mL/min/1.73 m^²^ compared with placebo-ACEi, with an intraindividual standard deviation of 17.5 mL/min/1.73 m^²^. With a 2-sided test with α=0.05 and >80% power, the sample size would have been 34 patients with hyperfiltration. Because of the unexpectedly low proportion of patients with hyperfiltration in the recruited cohort, study recruitment was stopped before reaching the planned sample size. Whereas the original hypothesis, related to the primary end point, was planned to be tested on the population with hyperfiltration as the primary analysis, this was not possible. However, a secondary analysis, prespecified in all versions of the protocol, was conducted on the overall trial population. This analysis was secondary in nature, but exploratory in relation to the predefined hypothesis regarding hyperfiltration. Secondary analyses were not part of the power or sample size calculations.

A random-effects crossover model, which is a mixed-effects model for repeated measures for a crossover design using restricted maximum likelihood, was used. The model uses a random effect for patient, fixed effects for the class variables treatment and period, and a fixed effect for the continuous variable randomization baseline of the end point. The model structure also gives rise to an implicit compound symmetry covariance structure for between-patient variance and consequently within-patient and between-patient variance is assumed to be independent under this model. The Kenward-Roger approximation was used to estimate denominator degrees of freedom and adjust standard errors. Significance tests were on the basis of least-squares means using a 2-sided α=0.05 (2-sided 95% CIs).

## Results

### Baseline Clinical and Anthropometric Characteristics

During the trial, only 2 of the 30 included patients (all with T1D) exhibited baseline hyperfiltration (GFR ≥135 mL/min/1.73 m^²^). Thus, because of the unexpectedly low proportion of patients with hyperfiltration, the study recruitment was stopped before recruitment of the planned sample size and analysis of patients with hyperfiltration compared with normofiltration was not pursued, and no patients with T2D or obesity only were recruited. The study population therefore comprised a homogeneous group of 30 patients with T1D (Table 1). Overall, patients had a mean age of 26.7±4.5 years with a diabetes duration of 16.0±7.0 years. Of the 30 included patients, 43.3% were male. This group of patients had normal mean estimated GFR (eGFR) of 121±12 mL/min/1.73 m^²^, normal SBP of 109±9 mm Hg, DBP of 68±5 mm Hg, and heart rate of 72±14 bpm. There were no patients with albuminuria.

**Table 1.**
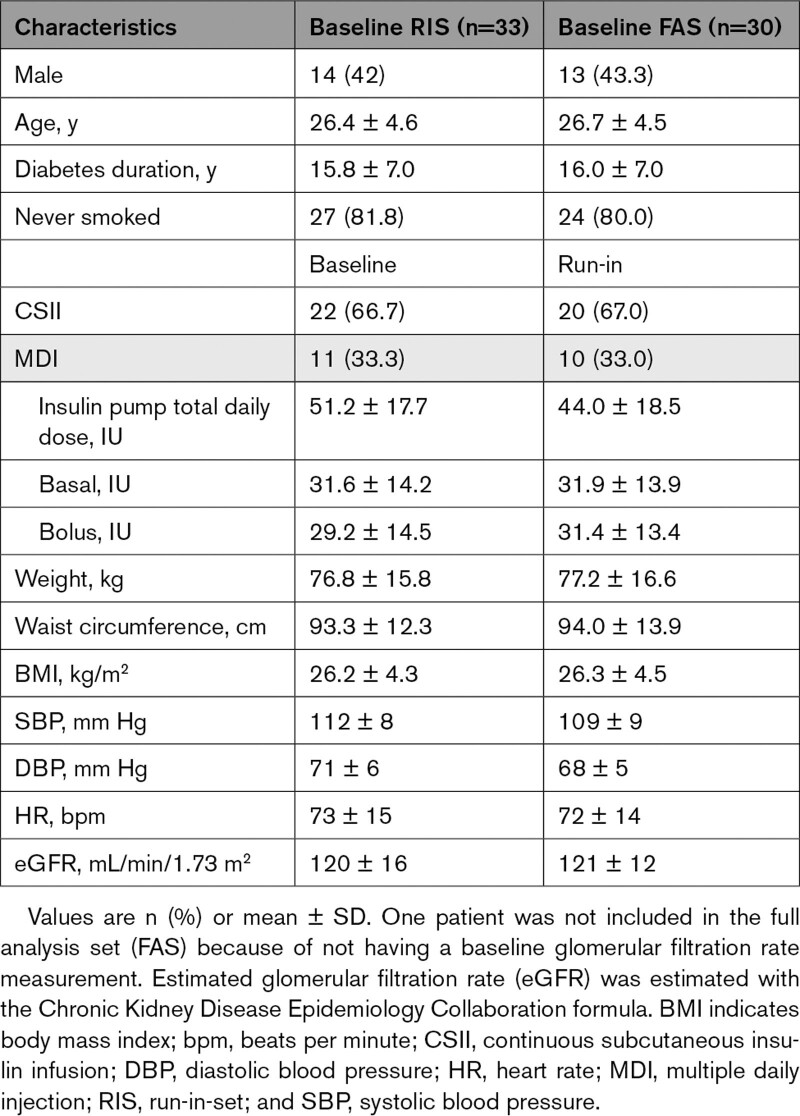
Clinical Characteristics at Screening Baseline and at Randomization Baseline After Run-In With Ramipril in Patients With Type 1 Diabetes

### Effect of Empagliflozin in Combination With Ramipril on Renal Function and Sodium Handling

**Figure 2.**
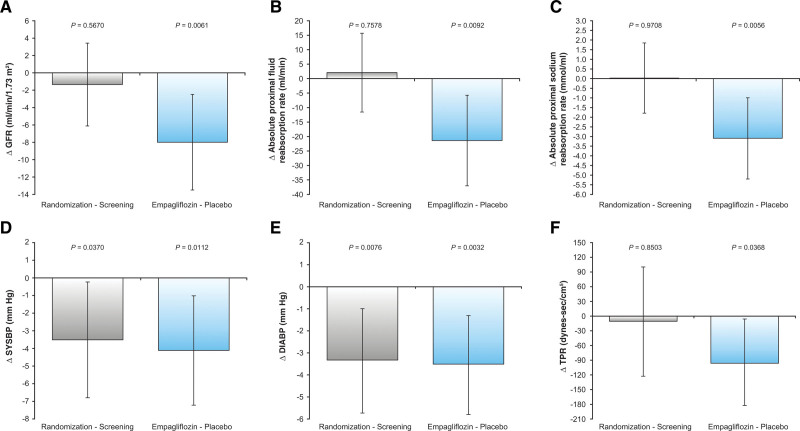
**Changes in glomerular filtration rate, absolute proximal fluid reabsorption rate, absolute proximal sodium reabsorption rate, systolic blood pressure, diastolic blood pressure, and total peripheral resistance during screening subtracted from randomization and placebo subtracted from empagliflozin in patients with type 1 diabetes at baseline and in response to ramipril treatment after addition of empagliflozin or placebo.** Changes in (**A**) glomerular filtration rate (GFR), (**B**) absolute proximal fluid reabsorption rate, (**C**) absolute proximal sodium reabsorption rate, (**D**) systolic blood pressure (SYSBP), (**E**) diastolic blood pressure (DIABP), and (**F**) total peripheral resistance (TPR) during screening subtracted from randomization and placebo subtracted from empagliflozin in patients with type 1 diabetes at baseline and in response to ramipril treatment after addition of empagliflozin or placebo.

Ramipril treatment for 4 weeks during the run-in period significantly decreased RVR (0.079±0.017 to 0.071±0.016 mm Hg/min/1.73 m^2^/mL; *P*=0.044). No other significant changes in the renal hemodynamic or tubular sodium handling measures were observed after ramipril treatment (Table 2). The addition of empagliflozin treatment for 4 weeks to the background of ramipril resulted in a significantly larger decrease in GFR of 5 mL/min/1.73 m^2^ (run-in ramipril: 115±12; 4 weeks added empagliflozin: 110±3 mL/min/1.73 m^2^) compared with an increase of 3 mL/min/1.73 m^2^ with placebo (run-in ramipril: 115±12; 4 weeks added placebo: 118±3 mL/min/1.73 m^2^; *P*=0.0061; Figure 2). There were no significant differences observed between the addition of empagliflozin or placebo to ramipril on other renal hemodynamic measures reported, such as ERPF, filtration fraction, renal blood flow, and RVR (Table 2). The decrease in absolute proximal sodium and absolute proximal fluid reabsorption rates was larger when empagliflozin was added to ramipril compared with placebo with ramipril (*P*=0.0056 and 0.0092, respectively; Table 2). Fractional sodium and lithium excretion was greater with addition of empagliflozin compared with placebo (*P*=0.030 and 0.008, respectively). There were no other significant differences in tubular sodium handling measures observed between the 2 groups.

**Table 2.**
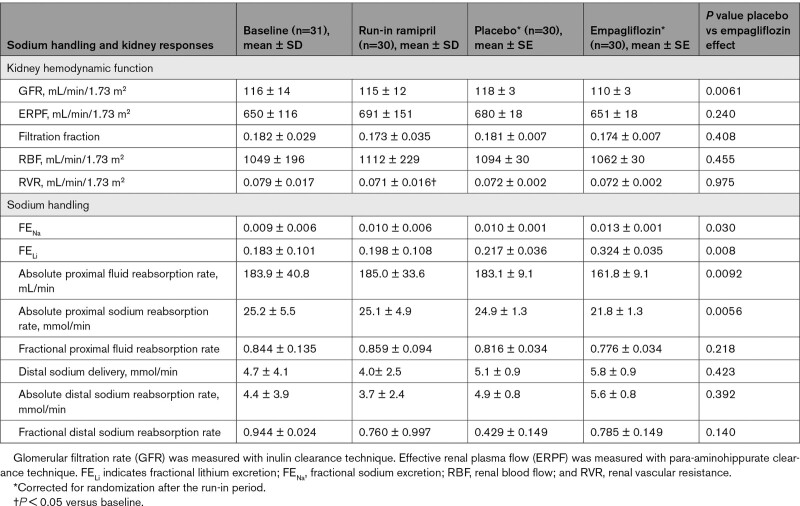
Sodium Handling and Kidney Hemodynamic Responses to Empagliflozin Compared With Placebo in Patients With Type 1 Diabetes

### Effect of Empagliflozin in Combination with Ramipril on Cardiovascular Hemodynamic Measures

Ramipril treatment for 4 weeks during the run-in period significantly decreased SBP, DBP, and mean arterial pressure clinic measurements, significantly decreased ABPM daytime and awake time measurements for SBP and DBP, and decreased DBP during sleep time on ABPM (Table 3). Ramipril treatment significantly decreased carotid augmentation index but did not affect any of the NICOM measurements.

**Table 3.**
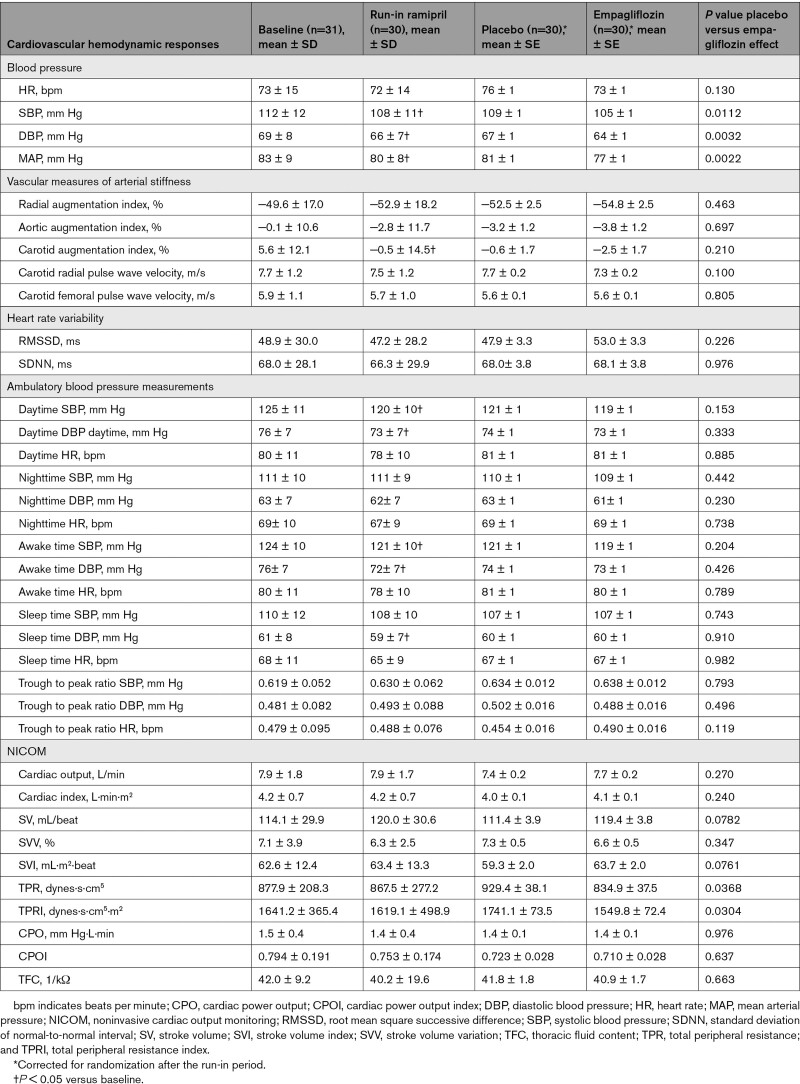
Cardiovascular Hemodynamic Responses to Empagliflozin Compared With Placebo in Patients With Type 1 Diabetes

The addition of empagliflozin treatment for 4 weeks to the background of ramipril resulted in additional declines in SBP, DBP, and mean arterial pressure compared with placebo (*P*=0.0112, 0.0032, and 0.0022, respectively; Figure 2). The total peripheral resistance decreased significantly with empagliflozin treatment compared with placebo (*P*=0.0368). There were no significant differences observed between the addition of empagliflozin or placebo to ramipril on other measures of the NICOM, arterial stiffness, heart rate variability, or ABPM outcomes.

### Effect of Empagliflozin in Combination With Ramipril on Metabolic Characteristics, Plasma, and Urine Biochemistry

Ramipril treatment for 4 weeks during the run-in period did not significantly change weight, waist circumference, or fasting plasma glucose level (Table 4). The addition of empagliflozin to ramipril decreased HbA1c by 0.4%, which was significantly greater compared with placebo (*P* < 0.0001). There were no significant differences in weight, waist circumference, or fasting plasma glucose level changes between the empagliflozin and placebo groups.

**Table 4.**
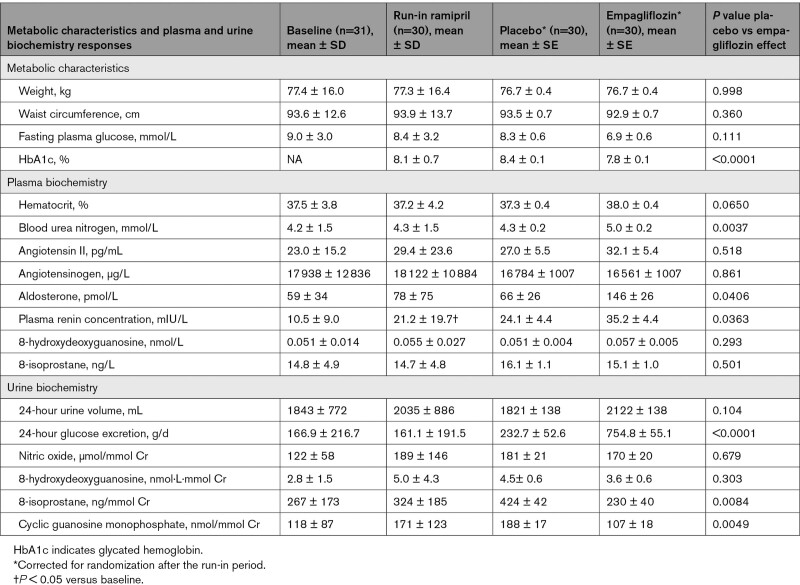
Metabolic Characteristics and Plasma and Urine Biochemistry Responses to Empagliflozin Compared With Placebo in Patients With Type 1 Diabetes

**Figure 3.**
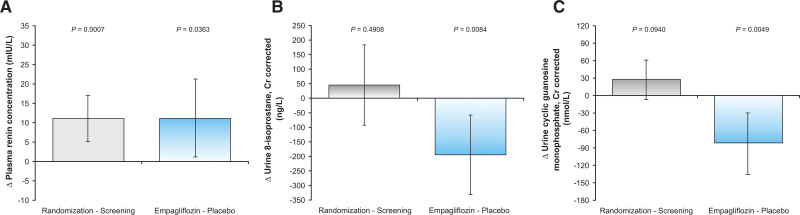
**Changes in plasma renin concentration, urine 8-isoprostane, and urine cyclic guanosine monophosphate during screening subtracted from randomization and placebo subtracted from empagliflozin in patients with type 1 diabetes at baseline and in response to ramipril treatment after addition of empagliflozin or placebo.** Changes in (**A**) plasma renin concentration, (**B**) urine 8-isoprostane, and (**C**) urine cyclic guanosine monophosphate during screening subtracted from randomization and placebo subtracted from empagliflozin in patients with type 1 diabetes at baseline and in response to ramipril treatment after addition of empagliflozin or placebo.

Ramipril treatment for 4 weeks increased plasma renin concentration (Figure 3), without significant changes to other plasma or urine biochemistry markers. A significantly larger increase in blood urea nitrogen and plasma renin was observed when empagliflozin was added to ramipril compared with placebo. A significantly larger increase in 24-hour glucose excretion was observed with empagliflozin compared with placebo. The increase in hematocrit in the SGLT2i-treated group did not reach statistical significance. Levels of 8-isoprostane and cyclic guanosine monophosphate decreased significantly more with empagliflozin compared with placebo. There were no other significant differences observed in the plasma or urinary biochemistry outcomes between the treatment groups.

### Adverse Events

Two serious adverse events occurred during the study, both during the placebo treatment phase: intestinal obstruction requiring surgery and testicular rupture attributable to an unrelated sports injury. Urinary tract infections occurred in 1 patient in each group (placebo, 3.3%; empagliflozin, 3.2%). A fungal infection and a vulvovaginal mycotic infection were also reported during empagliflozin treatment in 1 patient each (3.2%). Ketosis (β-hydroxybutyrate >1.5 mmol/L) occurred in 6 patients (19.4%) during empagliflozin treatment and in 1 patient (3.3%) during placebo treatment. There were no episodes of ketoacidosis during placebo or empagliflozin treatment. Hypoglycemia occurred in 1 patient (3.3%) and only during placebo treatment, but not during empagliflozin treatment. Dizziness and thirst occurred in 2 patients (6.5%), each during empagliflozin treatment. Hypotension was reported in 1 patient during the empagliflozin treatment (3.2%). Aside from dysuria in 1 patient (3.2%) during empagliflozin treatment, there were no other reported adverse events related to renal function. The overall incidence of reported adverse effects for the 31 patients who took study drug is reported in Table 5.

**Table 5.**
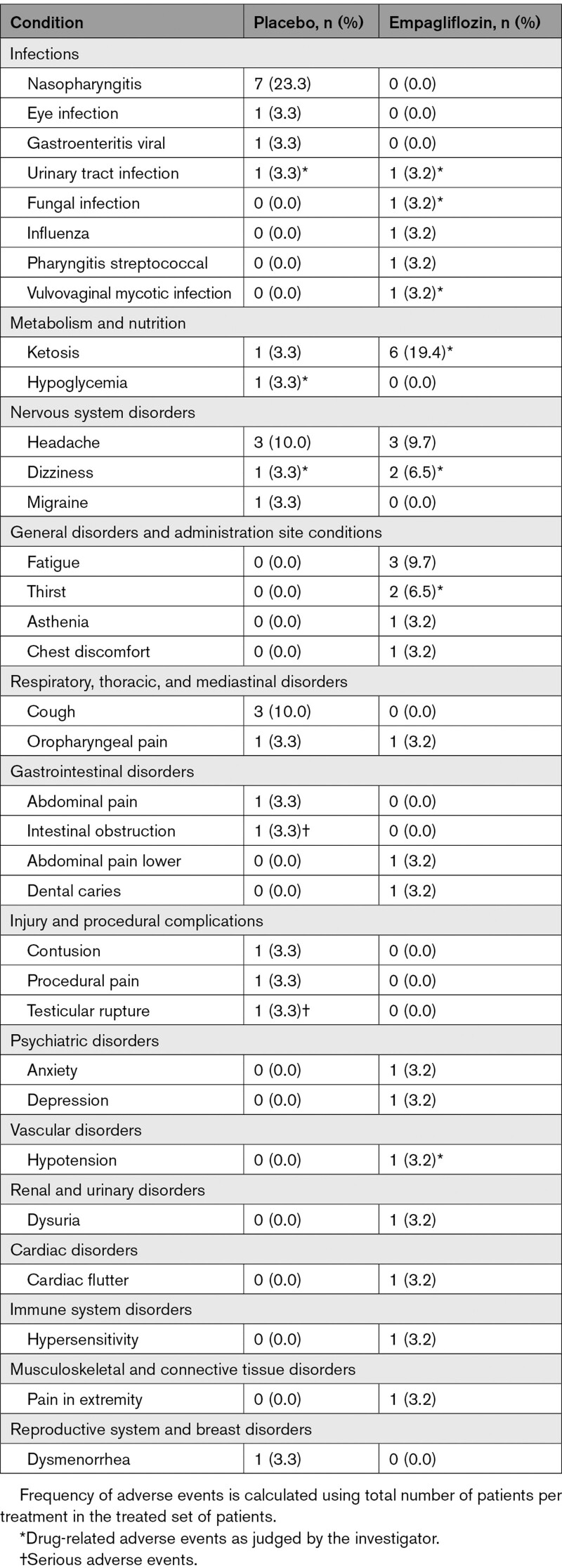
Adverse Events in Patients With Type 1 Diabetes During the Study Period

## Discussion

Combination SGLT2i-ACEi therapy in animal models of T2D was shown to result in additive nephroprotective effects, including reductions in blood pressure, proteinuria, glomerular injury, and renal fibrosis that were greater than those observed with the use of either drug class alone.^13^ Animal data are supported by observations in dedicated cardiovascular and renal outcome trials, where a large proportion of patients with T2D are on a background of RAASi. In these trials, SGLT2i also exhibited significant additive cardiorenal protection.^1–3^ The original aim of this study was to determine whether combined blockade of neurohormonal (RAASi) and tubular (SGLT2i) factors will reduce hyperfiltration and lead to additive beneficial effects in patients with renal hyperfiltration versus patients with normofiltration. The small number of recruited patients with hyperfiltration (n=2) precluded a direct comparison between patients with normofiltration and hyperfiltration. Nevertheless, our findings in this mechanistic study, as related to patients with T1D and preserved kidney function, will help to improve the understanding of the physiologic interaction between SGLT2i and RAASi and its effect on cardiorenal function. SGLT2i therapies are not approved for clinical use in patients with T1D in most countries. The totality of data generated in our cohort of uncomplicated patients with T1D serves as a mechanistic model without confounding comorbidities to help clarify the early renal and cardiovascular changes in diabetes or obesity and help guide research to address pathophysiologic dysregulations and treatment modalities in this context.

Our first major observation was that GFR decreased modestly with the addition of empagliflozin to ramipril treatment in our cohort of patients with T1D under euglycemic clamp conditions. Whereas pretreatment with ramipril alone decreased RVR, adding empagliflozin did not result in further RVR changes, suggesting that SGLT2i may act by mechanisms other than increasing afferent renal arteriole resistance in patients without hyperfiltration. SGLT2i blocks sodium reabsorption at the proximal tubule, as was evident with the decreased absolute proximal sodium and fluid reabsorption rate and increased fractional sodium and lithium excretion, which was not observed with RAASi. Therefore, our data uniquely identify that SGLT2i and RAASi target separate pathologic mechanisms in the kidney and may be used in combination for a synergistic effect on renal hemodynamic function.

Our findings also have important safety implications. In patients with normal GFR, kidney function was maintained with SGLT2i when combined with RAASi. In our previous study, GFR was reduced with SGLT2i only in patients with T1D and hyperfiltration, but not in those with normofiltration.^27^ Our findings are further supported by the RED study in 44 patients with T2D and preserved kidney function treated with SGLT2i on the background of RAASi.^28^ Similar to our observations, SGLT2i treatment in the RED cohort resulted in a decrease in GFR without significant changes to renal blood flow or RVR, suggesting a lack of effect on preglomerular factors. Therefore, whereas the effect of SGLT2i on afferent renal tone is evident in hyperfiltration, the mechanisms of SGLT2i in people with longer duration of diabetes and normofiltration may differ. In the RED study, the release of prostaglandins with SGLT2i was hypothesized to vasodilate the efferent renal arteriole in the context of afferent renal arteriole vasoconstriction, thereby leading to overall maintenance of kidney perfusion. Balanced preglomerular and postglomerular factors might also explain why SGLT2i do not induce acute kidney injury in large T2D trials, but instead induce their effects by a dose-dependent mechanistic eGFR dip as a result of renal hemodynamic changes over a wide range of GFR values, followed by a greater preservation of renal function over time.^29–31^ Even though the mechanism responsible for changes in GFR may differ in people with hyperfiltration compared with those without hyperfiltration, the acute hemodynamic change in GFR—likely reflective of reduced glomerular pressure—is consistent across T1D, T2D, and nondiabetic chronic kidney disease.^28,32^ On the basis of our current findings, this GFR dip also occurs in people with T1D who are already taking a standard of care RAASi.

Whereas the mechanisms responsible for GFR reduction with SGLT2i remain incompletely understood, a significant decrease in urinary 8-isoprostane was observed when empagliflozin was added to ramipril treatment, suggesting a decrease in renal oxidative stress. Combination treatment also significantly decreased cyclic guanosine monophosphate, the downstream signaling molecule of NO, a neurohormonal factor that contributes to preglomerular vasoconstriction. Animal models suggest that NO is released during tubuloglomerular feedback activation, contributing to a decrease in vasoconstriction^33,34^ and therefore pharmacologic inhibition of NO synthase in humans reduces GFR by a modest 10 to 12 mL/min/1.73 m^2^.^17,35,36^ In patients with uncomplicated T1D treated with only empagliflozin, however, there were no significant changes to NO.^20^ A physiologic interaction between intrarenal RAAS and NO bioactivity may affect the effects of combination treatment observed in our cohort. For example, intrarenal RAAS activation inhibits neuronal NO synthase leading to exaggerated vasoconstriction mediated by tubuloglomerular feedback.^37^ Given that we did not observe an obvious effect on tubuloglomerular feedback or RVR, the relationship between NO bioactivity, oxidative stress, and the acute decrease in GFR when SGLT2i is added to RAASi merits further investigation.

Our second major observation was the additive reductions in SBP and DBP during clinic visits when empagliflozin was combined with ramipril, which is consistent with previous trials in patients with T2D.^38,39^ The reduction in blood pressure during the clinic visits was accompanied by a significant increase in blood urea nitrogen and a numerical nonsignificant increase in hematocrit. These changes are likely to reflect a mild hemoconcentration effect because these changes in clinical markers of hemoconcentration are most closely linked with plasma volume contraction rather than increased hematopoiesis.^40^ Furthermore, in our study cohort, aldosterone levels nearly doubled and an increase in plasma renin concentration was observed when SGLT2i was added to ACEi treatment. A rise in circulating RAAS mediators may be of particular importance in patients concomitantly treated with RAASi and may reflect both pharmacologic RAAS blockade and plasma volume contraction. It is less clear, however, whether a decrease in plasma volume contributed to blood pressure lowering because no changes to weight or thoracic fluid content were observed. Results of the DAPASALT trial in patients with type 2 diabetes with preserved kidney function showed that SGLT2i treatment for 2 weeks with dapagliflozin reduced blood pressure without affecting natriuresis or volume status.^41^ In our cohort of patients, the blood pressure lowering with the addition of empagliflozin to ramipril may have been related to an observed decline in total peripheral resistance.

In this study, treatment with ramipril alone decreased carotid augmentation index, and the addition of empagliflozin did not lead to a further reduction in arterial stiffness measurements. This finding contrasts with the decrease in arterial stiffness observed when patients with uncomplicated T1D are treated with an SGLT2i alone.^42^ Therefore, there may be a limitation to the decrease in arterial stiffness in patients with uncomplicated T1D, and once pretreated with RAASi, no further effect on stiffness may be anticipated.

Empagliflozin was generally well tolerated in this short-duration and well-controlled mechanistic trial, aside from 2 serious adverse events that occurred during placebo treatment and were deemed unrelated to therapy. Ketosis was reported in 6 patients with T1D during SGLT2i treatment without any incidents of diabetic ketoacidosis; this observation emphasizes the need for careful monitoring of ketosis and adequate diabetic ketoacidosis risk mitigation when patients with T1D are given an SGLT2i in context of well-controlled experimental settings.

The main limitation of our study is the lack of patients with hyperfiltration, whom we hypothesized would benefit most hemodynamically when SGLT2i was added to ACEi treatment. Although we observed a lowering of GFR, it is likely that this effect would have been even greater in patients with higher baseline GFR especially in the hyperfiltration range. The low incidence of hyperfiltration may be a result of improved glycemic control in clinical practice, although the mean baseline HbA1c of 8.1% was suboptimal. Moreover, our measurements were conducted under clamped euglycemia, which decreases the chance of exhibiting elevated GFR levels. Future work may instead focus on the effect of adding SGLT2i to ACEi treatment on hyperfiltration induced by hyperglycemia, which could more actively reflect ambient glycemic conditions in clinical practice, characterized by periodic hyperglycemia. We were also unable to examine the effect of adding SGLT2i to ACEi treatment on albuminuria because of the low burden of kidney disease; albuminuria was absent at baseline in the majority of patients. Although we recognize the limitations imposed by the small sample size, we minimized the potential effect of the small sample size by using a prestudy preparation phase to control for factors influencing our physiologic measurements, such as dietary sodium intake. Variability was also decreased by the crossover study design, in which each participant acted as his or her own control.

We have demonstrated mechanistically that adding SGLT2i to ACEi treatment is associated with a significant kidney hemodynamic effect characterized by a modest decrease in GFR that was not observed when adding placebo to ACEi. Adding SGLT2i to ACEi also led to an additive blood pressure–lowering effect, which may be mediated in part by a decline in total peripheral resistance. Our results are supportive of a cardiorenal protective physiologic mechanism for use of SGLT2i when added to ACEi to lower intraglomerular pressure and reduce cardiorenal risks in people with diabetes.

## Article Information

### Acknowledgments

The authors thank James Bell for statistical assistance during study design. Editorial assistance, supported financially by Boehringer Ingelheim, was provided by Paul Lidbury of Elevate Scientific Solutions during the preparation of this manuscript. The authors were responsible for all content and editorial decisions. Drs Lytvyn, Kimura, Soleymanlou, and Cherney researched data and wrote the manuscript. N. Peter performed statistical analysis. V. Lai researched data and Dr Perkins contributed to discussion and reviewed and edited the manuscript. All authors approved the final version of the manuscript.

### Sources of Funding

Boehringer Ingelheim and the Canadian Institutes of Health Research provided support for this study to Dr Cherney. Dr Cherney also is supported by a Department of Medicine, University of Toronto Merit Award, Diabetes Canada, the Heart and Stroke Richard Lewar Centre of Excellence, and the Heart and Stroke Foundation of Canada.

### Disclosures

Dr Lytvyn, V. Lai, J. Tse, and L. Cham have no conflicts of interest to disclose. N. Peter is a statistical consultant for Boehringer Ingelheim. Dr Perkins reports consultancy agreements with Abbott, Boehringer Ingelheim, and Insulet; receiving research funding from the Bank of Montreal and Novo Nordisk; receiving honoraria from Abbott, Insulet, Medtronic, Novo Nordisk, and Sanofi; and serving as a scientific advisor or member of Abbott, Boehringer Ingelheim, Insulet, and Sanofi. Drs Kimura and Soleymanlou are employees of Boehringer Ingelheim. Dr Cherney has received honoraria from Boehringer Ingelheim-Lilly, Merck, AstraZeneca, Sanofi, Mitsubishi-Tanabe, AbbVie, Janssen, Bayer, Prometric, BMS, Maze, CSL Behring, and Novo Nordisk and has received operational funding for clinical trials from Boehringer Ingelheim-Lilly, Merck, Janssen, Sanofi, AstraZeneca, and Novo Nordisk.

### Supplemental Material

Figures S1 and S2

Tables S1–S3

## Supplementary Material


