# Supplementary material for: Renal and Vascular Effects of Combined SGLT2 and Angiotensin-Converting Enzyme Inhibition
Source: Circulation. 2022 Jul 11;146(6):450–62. doi: 10.1161/CIRCULATIONAHA.122.059150 (PMC9354594; doi:10.1161/CIRCULATIONAHA.122.059150)

SUPPLEMENTAL MATERIAL

Table S1. Detailed study inclusion/exclusion criteria.

**Inclusion Criteria**	
1.	Male or female patients diagnosed with T1D ≥ 6 months prior to informed consent or T2D or non-diabetic obese patients
2.	For T1D patients: experienced insulin pump users for ≥ 3 months prior to the study and willing to use the same insulin pump for the duration of the study or be on multiple daily injections
3.	For patients with T1D or T2D: HbA1c of 6.5%-11.0%
4.	Ability to follow an established carbohydrate counting method and an insulin titration algorithm based on investigator recommendations
5.	Age ≥ 18 years
6.	Body Mass Index (BMI) ≥ 18.5 kg/m^2
7.	Estimated GFR ≥ 60 $ml/min/1.73m^2$
8.	Average blood pressure > 90/60 mmHg and ≤ 140/90 mmHg
**Exclusion Criteria**	
1.	For T1D patients: treatment with an antihyperglycemic agent (e.g. metformin, alpha-glucoside inhibitors, pramlintide, glucagon-like peptide receptor agonist) within 3 months or history of hypersensitivity
2.	Treatment with an SGLT2i within 30 days
3.	Severe hypoglycaemia that required emergency hospital treatment within 3 months prior to screening
4.	History of organ transplantation, cancer, severe gastroparesis, brittle diabetes or hypoglycaemia unawareness based on investigator judgement
5.	Occurrence of DKA within 3 months
6.	Acute coronary syndrome, stroke or transient ischemic attack within 3 months
7.	Indication of liver disease as measured by either alanine transaminase (ALT), aspartate transaminase (AST), or alkaline phosphatase (AP)
8.	Any concomitant medication known to interfere with RAAS activity and/or renal function based on investigator judgement
9.	Any contraindications to ramipril per local product monograph
10.	Bariatric surgery or other gastrointestinal surgeries that lead to unstable body weight, or use of anti-obesity drugs within 3 months prior to screening
11.	Treatment with systemic corticosteroids or planned initiation of such therapy at screening
12.	Change in dose of thyroid hormones within 6 weeks or planned change at screening
13.	Blood dyscrasias or any disorders causing haemolysis or unstable red blood cells
14.	Pre-menopausal women who are pregnant, nursing, or who plan to become pregnant within the trial
15.	Alcohol or drug abuse within 3 months prior to informed consent that would interfere with trial participation or any ongoing clinical condition that would jeopardize subject safety or study compliance based on investigator judgement
16.	Participation in another trial with an investigational drug within 30 days prior to informed consent

Table S2. Parameters measured at each physiologic study visit categorized by cardiorenal mechanism of health and disease.

**Renal Hemodynamic Function**	**Systemic Hemodynamics (Non-Invasive Cardiac Monitoring)**
Glomerular filtration rate (GFR)<br>Filtration fraction (FF)<br>Renal blood flow (RBF)<br>Renal vascular resistance (RVR)	Cardiac output<br>Cardiac power output index<br>Stroke volume<br>Total peripheral resistance<br>Total peripheral resistance index<br>Thoracic fluid content
**Renal Sodium Handling**	**Metabolic Parameters**
Na Clearance (C_{Na})<br>Li Clearance (C_{Li})<br>Absolute "proximal" fluid reabsorption rate (APR)<br>Absolute "proximal" Na reabsorption rate (APR_{Na})<br>Fractional "proximal" fluid reabsorption (FPR)<br>"Distal" Na Delivery (DD_{Na})<br>Absolute "Distal" Na Reabsorption rate (ADR_{Na})<br>Fractional "Distal" Na Reabsorption (FDR_{Na})<br>$FE_{[electrolyte]}$	HbA1c<br>Fasting plasma glucose<br>Weight<br>Waist circumference
**Cardiovascular Hemodynamic Function**	**Renin Angiotensin Aldosterone System Plasma Markers**
Ambulatory blood pressure monitoring (ABPM)<br>Mean arterial pressure (MAP)<br>Systolic blood pressure (SBP)<br>Diastolic blood pressure (DBP)<br>Heart rate (HR)	Angiotensin II<br>Angiotensinogen<br>Aldosterone<br>Plasma renin concentration
**Arterial Stiffness**	**Oxidative Stress**
Augmentation index<br>Pulse wave velocity (PWV)<br>Heart rate variability (HRV)	8−hydroxydeoxyguanosine plasma and urine<br>8−isoprostane plasma and urine<br>Nitric oxide (NO) urine<br>Cyclic guanosine monophosphate (cGMP) urine

Table S3. Detailed study outcome calculations.

**Renal Hemodynamic Function**	
Filtration fraction (FF)	$\frac{GFR}{ERPF}$
Renal blood flow (RBF)	$\frac{ERPF}{1 - Hematocrit}$
Renal vascular resistance (RVR)	$\frac{MAP}{RBF}$
**Renal Sodium Handling**	
Na Clearance (C_{Na})	$\frac{[Urine\ Na]\ \times\ Urine\ flow\ rate}{[Plasma\ Na]}$
Li Clearance (C_{Li})	$\frac{[Urine\ Li] \times Urineflow\ rate}{[Plasma\ Li]}$
Absolute "proximal" fluid reabsorption rate (APR)	GFR – C_{Li}
Absolute "proximal" Na reabsorption rate (APR_{Na})	Plasma Na x APR
Fractional "proximal" fluid reabsorption (FPR)	$\frac{APR}{GFR}$
"Distal" Na Delivery (DD_{Na})	Plasma Na x C_{Li}
Absolute "Distal" Na Reabsorption rate (ADR_{Na})	DD_{Na} – (Urine Na x Urine flow rate)
Fractional "Distal" Na Reabsorption (FDR_{Na})	$\frac{ADR_{Na}}{DD_{Na}}$
$FE_{[electrolyte]}$	$\frac{[urine\ electrolyte] \times [plasma\ creatinine]}{[plasma\ electrolyte] \times [urine\ creatinine]}$

Figure S1. Flow diagram of study participants.

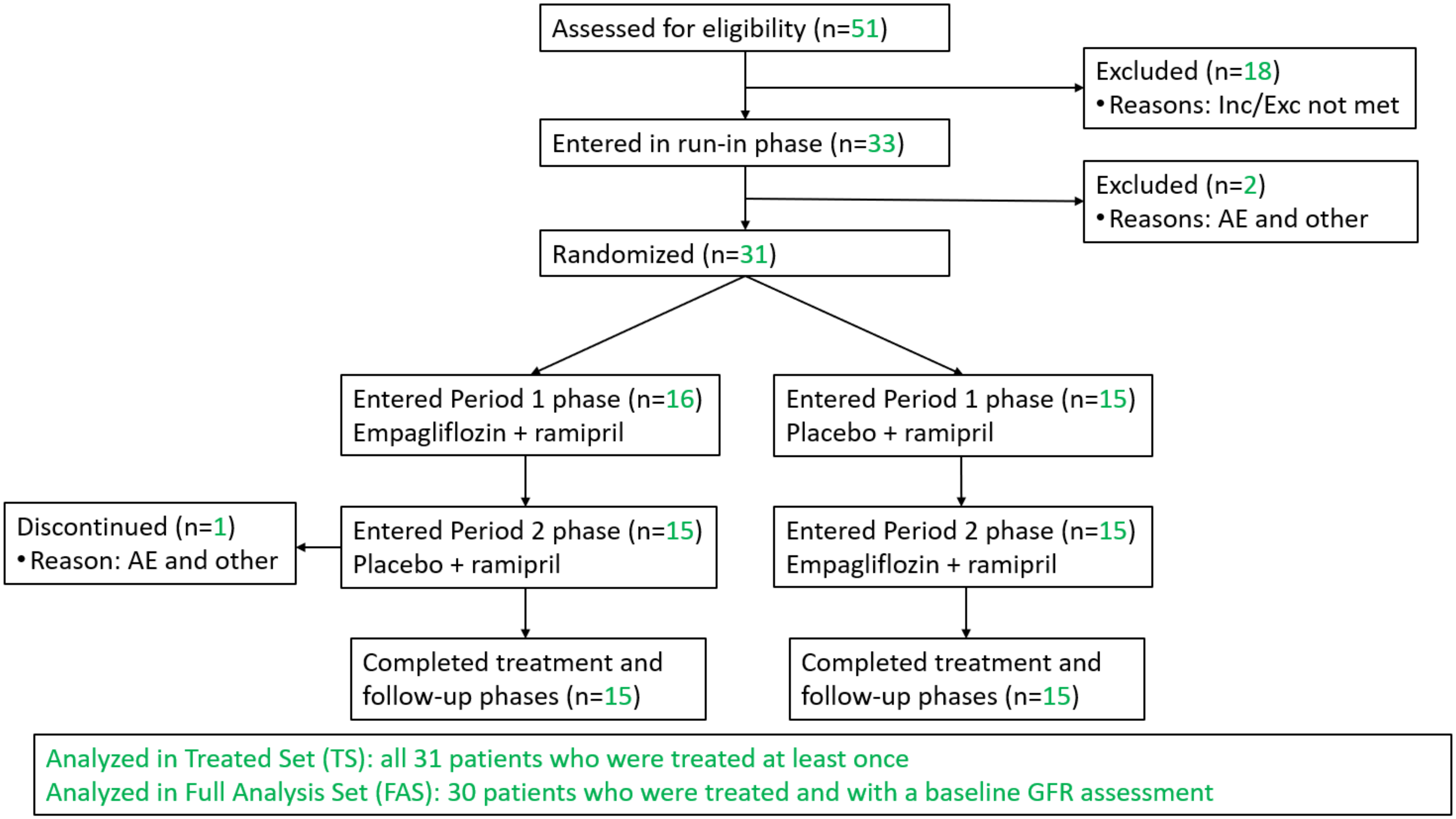

Figure S2. Study design for kidney hemodynamic function tests.

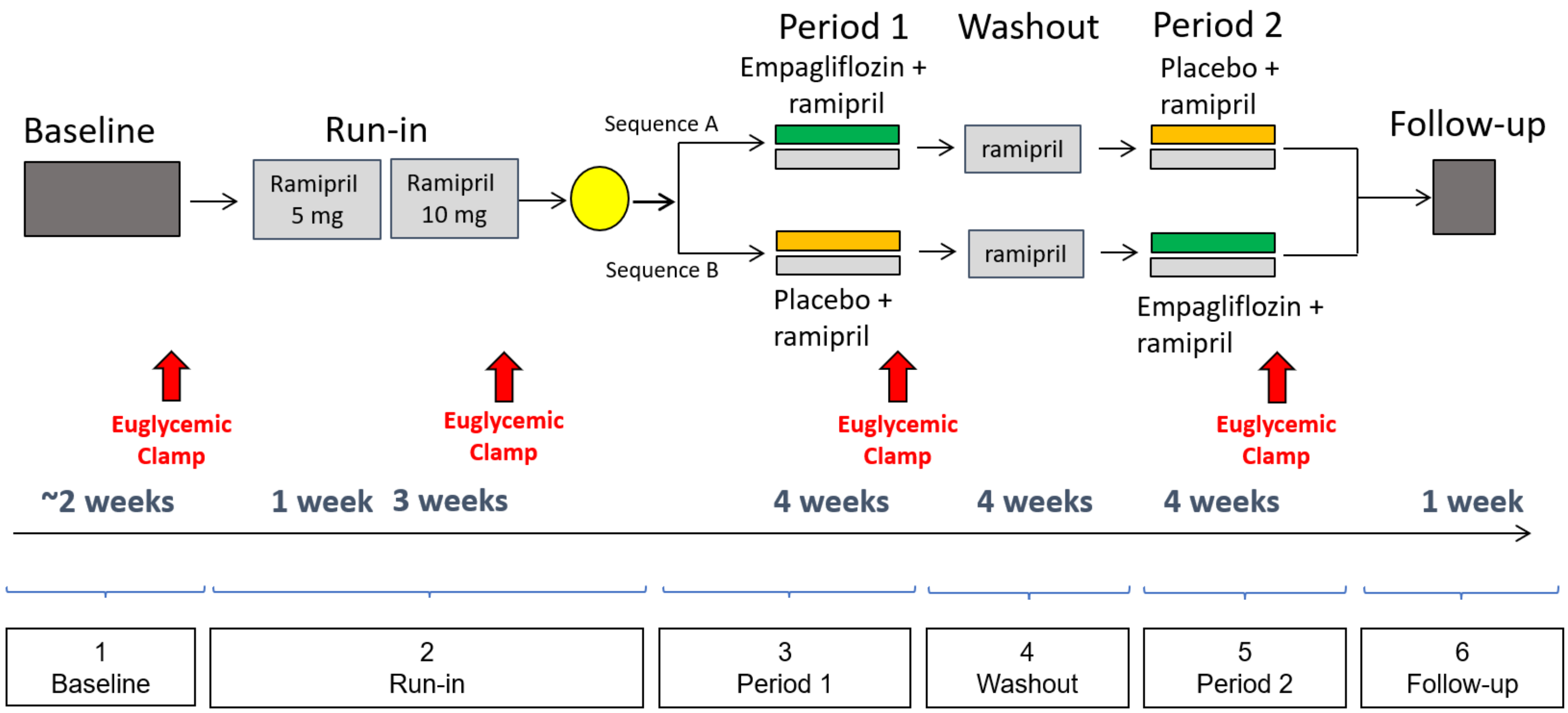

Supplement: Supplementary file 1 [file cir-146-450-s001.pdf]
